# Global Health Interest Among Pediatric Residents in Indiana Following the COVID-19 Pandemic: A Descriptive Study

**DOI:** 10.7759/cureus.88767

**Published:** 2025-07-25

**Authors:** Palka R Patel, Scott L Coven, Mitali S Thanawala, Feenalie Patel, Christina E Knight, Melissa R Thomas, Shaina M Hecht, Bobbi J Byrne, Megan S McHenry

**Affiliations:** 1 Department of Pediatrics, Indiana University School of Medicine, Indianapolis, USA; 2 Department of Medicine, Indiana University School of Medicine, Indianapolis, USA; 3 Division of Pediatric Hematology, Oncology, and Stem Cell Transplant, Riley Hospital, Indianapolis, USA

**Keywords:** covid-19, global health, medical education, medical residency, pediatrics

## Abstract

Introduction: While COVID-19 created unique learning experiences within medical education, the pandemic also changed views on global health (GH). There is an important gap in our knowledge regarding the ways in which the unique learning experiences of the COVID-19 pandemic affected medical residents’ approach to GH. Our objective is to understand the impact of the COVID-19 pandemic on pediatric graduate education, with special attention given to resident perspectives on GH education.

Methods: As part of a program-wide assessment of GH education in March 2021, a 50-item survey was developed based on widely used survey instruments, with additional questions related to COVID-19. In this analysis, we reviewed 13 quantitative questions assessing pediatric residents’ perceptions of how the COVID-19 pandemic affected their GH education. The electronic survey was sent via email to all pediatric and combined pediatric residents. Survey participation was voluntary. The questions were administered through REDCap™ (Research Electronic Data Capture, Vanderbilt University, Nashville, TN), and results were assessed using descriptive statistics, including percentages, frequencies, and distribution of responses, via Microsoft Excel (Microsoft Corporation, Redmond, WA). This assessment received exempt approval on ethics review.

Results: Of the 158 eligible pediatric residents, 96 (61%) completed the survey. Most residents expressed that the pandemic had a negative effect on GH education, scholarly activity, and residency training. When asked how their interest in GH changed during the pandemic, residents reported increased interest in learning about international advocacy and equity topics such as vaccine distribution (62/96, 65%), global disease spread (52/96, 54%), health disparities (49/96, 51%), and inequitable distribution of global resources (52/96, 54%). Interest in international electives varied depending on the presence of long-term GH intentions, while interest in domestic GH experiences remained unchanged (76/96, 79%) for most residents.

Conclusions: Although pediatric trainees reported that their GH education and international electives were negatively affected by the COVID-19 pandemic, many expressed increased interest in GH topics directly related to the pandemic. Graduate medical training programs can leverage current GH events to provide education that residents find both valuable and relevant.

## Introduction

The COVID-19 pandemic adversely affected graduate medical training, with trainee redeployment to areas outside their specialty, schedule adjustments to limit exposure, and the postponement or cancellation of routine medical care [1-3]. Pediatric residency programs noted adverse effects on trainees’ inpatient, outpatient, and procedural competence, and their preparation for senior roles [4]. Many didactic and other educational sessions were transitioned to remote or virtual learning, which may have impacted learning styles, group interaction, and trainee preferences [5,6]. For physician trainees interested in global health (GH), the COVID-19 pandemic simultaneously disrupted international learning experiences and revealed challenges in global public health [3,7].

Learning about GH-related topics has important relevance for pediatrics, even before the COVID-19 pandemic. Pediatric residency programs are increasingly offering training in GH and international child health topics. The American Board of Pediatrics recommends incorporating aspects of GH into the education of all pediatric trainees [8], and the Accreditation Council for Graduate Medical Education outlines, in its program requirements for pediatrics, the importance of competence in cultural humility and in interpersonal and communication skills, including communicating across diverse cultural and language backgrounds [9]. Additionally, GH training can improve residents’ ability to identify and care for patients who may have a unique set of needs because of their cultural or migration background [10,11].

To strengthen GH training, our pediatric residency program conducted a needs assessment to evaluate the existing GH curriculum among trainees. This assessment coincided with the COVID-19 pandemic, which led to the transition of in-person educational sessions to remote didactic sessions, morning reports, grand rounds, and noon conferences, as well as limited clinical exposure and the cancellation of international electives for our program. We hypothesized that witnessing a global pandemic also allowed residents to develop new perspectives on global disease. While the overall goal of our survey was to conduct a needs assessment of the current GH curriculum within our pediatric residency program, few studies at the time described pediatric resident perspectives on the impact of COVID-19 on GH education. Therefore, questions related to trainee perceptions of the educational impact of COVID-19 were added to our survey. Our objective was to assess changes in trainee attitudes and interest in GH education and opportunities before, during, and after their experience of residency training in the COVID-19 pandemic at a large Midwestern pediatric training program.

## Materials and methods

Setting and participants

This cross-sectional study took place at a large free-standing children’s hospital in Indiana with a robust GH education curriculum. All residents in the categorical and combined pediatric training programs were eligible and targeted for recruitment from March 15 to 27, 2021. Participation was voluntary.

All eligible residents in this study participated in the general pediatrics curriculum, which includes GH education designed for all pediatric trainees, as well as the option for additional GH training. Core GH educational activities include didactic sessions during noon conferences and grand rounds; a community-based elective focused on local resources; small-group sessions addressing pediatric GH learning objectives; and experiential learning such as simulation exercises [12,13], interpreter shadowing, and clinical exposure in a refugee clinic. Optional opportunities for residents with a deeper interest in GH include domestic and international GH electives and participation in the interdisciplinary GH pathway (formerly known as the GH track).

Interventions

The full survey used for this study included modified items from prior surveys of pediatric residency GH education and new questions related to coronavirus disease 2019 (COVID-19). Developed as part of a program-wide assessment of GH education, the survey was modified from a Wisconsin GH needs assessment and GH-focused questions from the widely used American Academy of Pediatrics (AAP) graduating residency questionnaire [14,15]. The Wisconsin assessment was conducted among primary care residents in 2007 and validated by several faculty members, while the annual AAP graduating residency questionnaire is pilot-tested by pediatric residents, developed by experienced researchers, and based on a thorough review of the literature. The full survey includes both quantitative and qualitative questions.

Because of the novelty of the COVID-19 pandemic at the time of survey administration, there were no previously validated surveys assessing the impact of COVID-19 on GH education in the target population. As such, of the 50 modified items in the full survey, 13 quantitative questions were developed by the study team to assess the impact of COVID-19 and resident interest in GH education and training activities during and before the pandemic. 

Before being administered, the full survey was iteratively reviewed and edited by faculty with experience in survey development (MM, SC) in collaboration with the wider study team (Supplemental Content 1). The questions, including the COVID-19-related survey items, were reviewed and edited by faculty and experienced pediatric educators for face and content validity using qualitative methods only [16]. Additionally, three pediatric residents (FP, CEK, MST) provided feedback on the survey as part of the survey development team before it was distributed to the wider pediatric resident cohort.

Outcomes measured

Survey questions evaluated participant demographics, and residents were asked to rank their interest in specific GH topics on a Likert scale, indicate their individual GH experiences, and state their level of commitment to future international activities. Residents were asked to rank their residency program on a five-point Likert scale regarding current preparedness to address GH topics. We also assessed exposure to GH curricular components during and before residency training.

Residents were asked to rank the impact of COVID-19 on their interest in GH experiences on a Likert scale as “increased,” “decreased,” or “unchanged.” The questionnaire ascertained the reasons residents indicated intent to pursue an international or domestic elective course, with options for open-ended answers. Three questions asked residents to rate the impact of the COVID-19 pandemic on their overall residency education using a five-point Likert scale. Anonymized individual-level data can be made available upon request.

Analysis of the outcomes

Data were collected and stored in a secure REDCap™ (Research Electronic Data Capture, Vanderbilt University, Nashville, TN) database [17]. From the REDCap™ database, data were exported to Excel (Microsoft Corporation, Redmond, WA) and cleaned, then questions relating to the impact of COVID-19 were analyzed using descriptive methods of statistical analysis, such as percentages, frequencies, variability, and distribution of responses. Likert-type questions were dichotomized as agreeing with (indicating “significant,” “moderate,” or “slight” impact) and disagreeing with (indicating “positive” or “no impact at all”) statements of adverse COVID-19 impact. No additional statistical software was used to analyze the data.

Ethics statement

The Institutional Review Board at Indiana University provided exempt approval for this study (Protocol #10479). Participants completed electronic assent via REDCap™ before beginning the full survey, and a $5 gift card was provided upon completion.

## Results

Demographics

Of the 158 eligible categorical and combined residents, 71% (112/158) initiated and 61% (96/158) completed the survey. The majority of respondents were female (71%, 68/96) and in their second or third year of residency (65%, 63/96). Roughly half of the respondents were categorical residents (53%, 51/96), followed by medicine-pediatrics residents (32%, 31/96), and one-quarter were formally part of the interdisciplinary GH track (25%, 24/96). Overall, in three areas of education (i.e., GH education, scholarly activity, and residency education), the majority agreed that COVID-19 had an adverse effect on their residency (see Table 1).

**Table 1:** Demographics of Respondents PGY: postgraduate year *Agreed that there was a significant adverse impact, moderate adverse impact, or slight adverse impact from COVID-19

Total Respondents	n (%)
Training level (N=96)	
PGY1	25 (26)
PGY2	30 (31)
PGY3	33 (34)
PGY4+	8 (8)
Gender (N=96)	
Female	68 (71)
Residency program (N=96)	
Categorical pediatrics	51 (53)
Combined internal medicine-pediatrics	31 (32)
Emergency medicine-pediatrics	2 (2)
Triple board (peds-psych-child psych)	7 (7)
Child neurology	3 (3)
Neurodevelopmental pediatrics	2 (2)
Within the global health track (N=96)	
Yes	24 (25)
Level of educational debt (including spouse) (N=94)	
>$100,000	67 (70)
Future career plans (N=96)	
Primary care	20 (21)
Subspecialty practice	61 (64)
Hospitalist	11 (11)
Other	4 (4)
Married/partnered (N=96)	
Yes	51 (53)
Have or are currently expecting children (N=96)	
Yes	15 (16)
Languages spoken/understood fluently (N=96)	
1 language	62 (65)
2 languages	26 (27)
3+ languages	8 (8)
Location of medical training (N=96)	
Other locations besides US or Canada	11 (11)
Lived outside the US for >1 year (N=96)	
Yes	26 (27)
Intention to participate in an international elective in the next academic year (N=96)	
Yes	45 (47)
Global health education has been adversely impacted by the COVID-19 pandemic* (N=95)	
Number of residents who agreed	70 (74)
Scholarly activity has been adversely impacted by the COVID-19 pandemic* (N=95)	
Number of residents who agreed	79 (83)
Residency education has been adversely impacted by the COVID-19 pandemic* (N=95)	
Number of residents who agreed	91 (96)

COVID-19 impact on GH education

**Figure 1:**
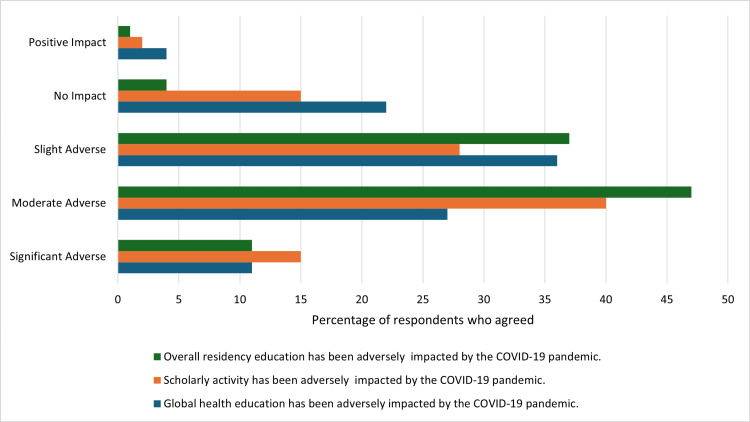
Residents’ Perceived Impact of COVID-19 on Overall Residency, Scholarly Activity, and Global Health Education (N=95)

There were diverse ranges of impact reported, with many residents reporting slight (36% for GH; 37% for residency), moderate (27%; 47%), or significant (11% for both) adverse effects of the pandemic on their GH education activities and overall residency experience, respectively. Only five respondents (5%) reported that COVID-19 had a positive or no impact on their residency, while up to 25 residents (26%) indicated that it had a positive or no impact on their GH activities (Figure 1).

Interest in GH topics before and after the pandemic

**Figure 2:**
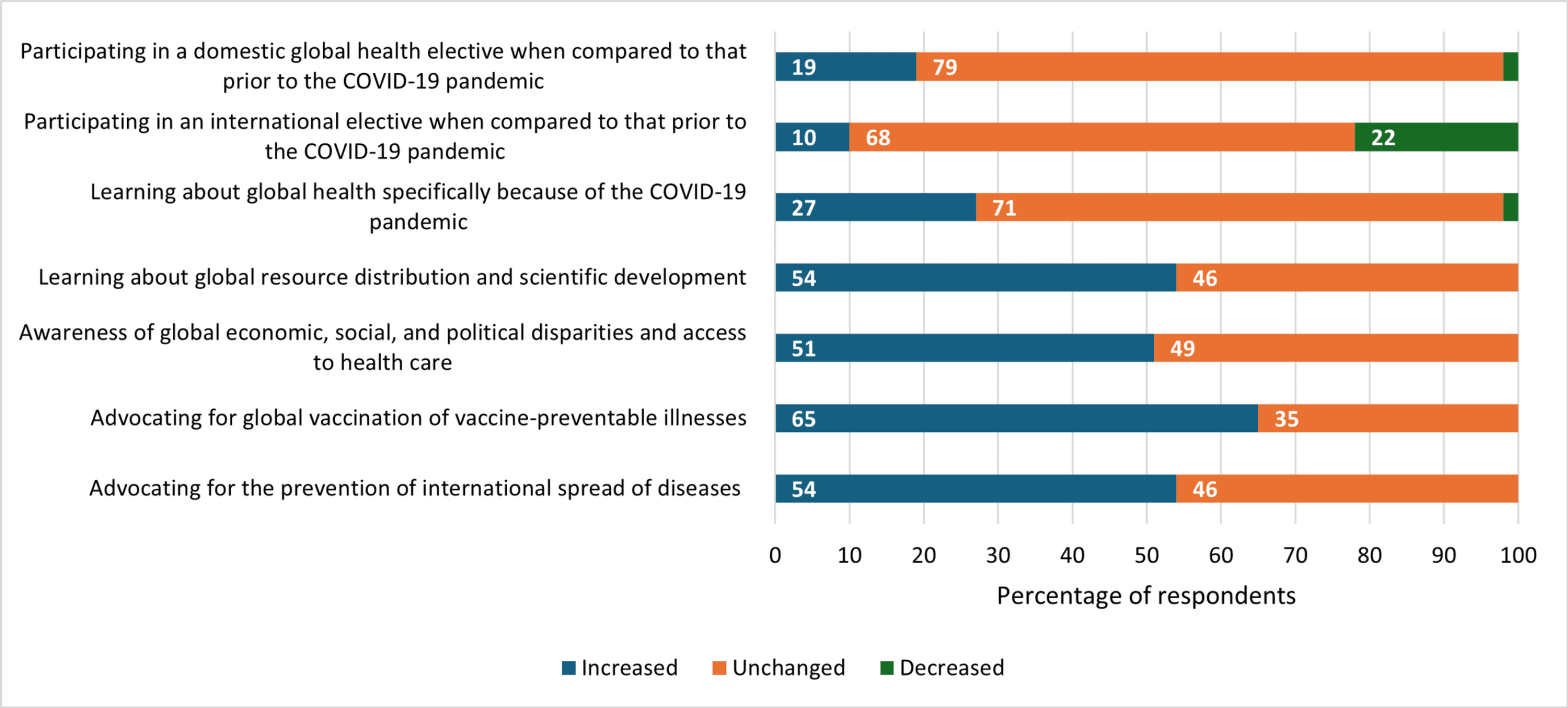
Changes in Resident Interest of Global Health Education Topics Following COVID-19 (N=96)

Most residents reported no change in interest in learning about GH (68/96, 71%) and in pursuing international electives (65/96, 68%) compared to prior to the pandemic (Table 3: Supplemental Content 2). COVID-19 decreased interest in traveling internationally for electives among 22% of trainees. However, 27% (26/96) reported increased interest in formally learning about GH, and 19% (18/96) expressed increased interest in domestic GH electives. Over half of the respondents reported increased interest in advocating for global vaccination (62/96, 65%), and increased learning about the social aspects of disease, such as health disparities (49/96, 51%) and equity in global resources (52/96, 54%) because of the pandemic (Figure 2).

Motivators and barriers for GH electives

Trainees interested in participating in future international electives (45/96, 47%) ranked long-standing interest in GH (37/45, 82%), the perception of the pandemic as a learning opportunity (33/45, 73%), and their COVID-19 vaccination status (22/45, 49%) most highly as their reasons (or motivators) for continued interest. For individuals not interested in an international elective (51/96, 53%), the most common reasons provided were lack of time (36/51, 71%), cost (23/51, 45%), and concern for missing training experiences (15/51, 29%). Compared to before the COVID-19 pandemic, about 20% of respondents indicated a decreased interest in international electives. When considering domestic GH electives, most residents (75/96, 78%) rated their interest unchanged when compared to pre-COVID-19, with a small number sharing an increased interest (18/96, 19%).

## Discussion

We aimed to assess the impact of COVID-19 on resident perceptions of changes in attitudes, interest, and opportunities for GH education among all pediatric residents. Our study indicated mixed results regarding the impact of COVID-19 on GH education and interest: residents reported that COVID-19 did not change their interest in learning about GH, while also negatively affecting their general residency education, scholarly activities, and GH electives and education. However, the majority of residents reported an increased interest in specific GH advocacy, knowledge, and awareness directly related to the COVID-19 pandemic. This suggests that, despite negative or neutral changes in interest in GH education, the COVID-19 pandemic may have spurred interest in action-oriented GH topics directly related to the pandemic, such as international vaccine advocacy and global sociopolitical disparities.

Findings in the current study show that residents had increased interest in learning about GH topics relevant to their clinical reality. The COVID-19 pandemic forced medical education in the US towards more introspective activities, which was in some ways negative for trainees [18-20]. Previous studies have assessed the effect of COVID-19 on medical education and personal well-being in trainees, showing improvements in work-life balance along with increased rates of stress and moral distress during the pandemic [19-25]. However, reflection and introspection during the pandemic also highlighted global and local inequities [26,27]. Pediatric residents are developing greater awareness of the role of diversity and health equity in health outcomes and the importance of learning to provide culturally responsive care, even beyond the context of COVID-19 [28]. The pandemic compounded this awareness by highlighting clinically relevant GH equity topics for trainees as they engaged in the changing realities of medical education and care provision [21]. Witnessing disparities and inequities worldwide may have adjusted trainees’ professional identity and resulted in increased exposure to the core tenets of GH [26,29]. Based on these results, our team aims to utilize current and relevant examples for GH education in the general pediatric training program and link the relevance of GH training topics to current clinical practice realities.

Literature exists regarding residents’ perceptions of the effect of the pandemic on general education [30,31]. Our study adds to the limited references that directly assess trainee perspectives on GH-specific education during the pandemic [32]. This study specifically identified increased interest in pandemic-relevant GH topics, with somewhat conflicting results regarding neutral change in interest for GH education overall. Based on these findings, we identified among our trainees the importance of not only asking about perceptions of GH in general but also including specific examples of GH topics. Future research may aim to determine pediatric residents’ perspectives on what GH encompasses, and regularly reassess pediatric residents’ GH interest and participation. Additional work conducted on a larger scale may be more generalizable than the current study and help direct national changes to GH education. Related to the effects of COVID-19 on GH education, other literature has provided a general overview of adapting GH courses during the transition to virtual education [3,33]. Despite the documented barriers to instituting sufficient GH programs [28,34], many appraisals have reviewed how GH work could be made more equitable or reimagined, especially in the context of COVID-19 [35-39]. While our study did assess the impact of the pandemic on trainee perspectives and plans to participate in domestic and international GH electives, it did not review the effects on our institution’s international partnerships.

Based on our findings, we recommend that residency programs continually assess the state of their existing GH education in the context of the residency core curriculum. Importantly, selected GH training topics should be made available to all trainees, not only siloed to those participating in GH training “tracks” or “pathways.” This ensures that individuals with a diverse range of interests in GH receive the benefits of learning about GH competencies, such as cultural humility training and changing disease patterns [40,41]. As programs look forward from the COVID-19 pandemic, thoughtfully designed GH education curricula tailored to trainee interests, community needs, and global realities can provide accessible and relevant GH training.

While this study elucidated unique findings about pediatric residents’ perspectives on GH education, there are limitations to consider, such as the lack of validated survey items related to COVID-19 and recall bias regarding self-reported past interests and experiences. Without existing perceptions from residents recorded before the pandemic, we recognize the limitations of relying on self-reported data. Given the small sample size at our single institution, which has an interest in GH education, our descriptive findings may not be generalizable. Additionally, it is unclear how our findings on resident interest in GH learning and education may change over time. Due to the structure of the survey instrument, descriptive methods rather than statistical analysis were employed to avoid overinterpretation of the results; this remains a limitation of the survey tool itself.

## Conclusions

The COVID-19 pandemic simultaneously disrupted typical medical training programs and highlighted the global interconnectedness of health and disease. Our study supports previous findings about the negative impacts of the pandemic on general residency education and adds to the current literature through findings that trainees reported increased interest in learning about and advocating for pandemic-specific GH topics. As we move forward from the lessons learned about GH during the COVID-19 pandemic, future studies are needed to explore how trainee perspectives over time regarding GH education, including larger sample sizes and more robust statistical analysis. By identifying GH topics that are meaningful and relevant to pediatric trainees, GH educators can ensure that curricular elements remain dynamic, inclusive, and relevant as a part of general pediatric residency education.

## Appendices

**Table 2:** Supplemental Content 1. Full Program-Wide Global Health Needs Assessment Tool

Part I: General Global Health Interest/Experiences
1. I am interested in learning about health inequities during residency.
A. Strongly Agree; B. Moderately Agree; C. Somewhat Agree; D. Somewhat Disagree; E. Moderately Disagree; F. Strongly Disagree
2. I am interested in learning about global health during residency.
A. Strongly Agree; B. Moderately Agree; C. Somewhat Agree; D. Somewhat Disagree; E. Moderately Disagree; F. Strongly Disagree
3. What experience(s) have you had in global health? (select all that apply)
A. Formal global health courses/lectures/conferences (e.g., within noon or case conference); B. Formal cultural competency/humility classes/lectures/conferences; C. Formal global health track/certificate/degree in training; D. University-approved international medical rotations/electives; E. Personal international medical/global health trip; F. Domestic clinical global health experiences (e.g., Indian Health Service, working at Marion County Refugee clinic, etc.); G. Have lived more than one year of your life in a country outside the US; H. Group discussion of the GOALS modules on Community Rotation (global health topics); I. Sim Olympic scenario (residency simulation day) that included a refugee or immigrant; J. Rotation or course focused on the social determinants of health; K. Shadowing an interpreter and writing a reflection
Part II: Residency Training Experiences
1. Are you in the IU interdisciplinary global health track?
A. Yes; B. No
2. If you were interested in one or more of the global health experiences from Part I, but were unable to participate during residency, please tell us what that/those experience(s) was/were and why? (free text)
3. What were the most enjoyable global health experiences you had during residency? (free text)
Part III: Selecting Residency Training
1. The interdisciplinary global health residency track at IUSM influenced your decision to rank IU higher for residency match.
A. Strongly Agree; B. Moderately Agree; C. Somewhat Agree; D. Somewhat Disagree; E. Moderately Disagree; F. Strongly Disagree
2. It was important for you to choose a residency program that had opportunities for a global health elective, regardless of whether there was a formal interdisciplinary global health residency track at IUSM.
A. Strongly Agree; B. Moderately Agree; C. Somewhat Agree; D. Somewhat Disagree; E. Moderately Disagree; F. Strongly Disagree
3. It was important for you to choose a residency program that had exposure to formal global health topics (courses, lectures, conferences).
A. Strongly Agree; B. Moderately Agree; C. Somewhat Agree; D. Somewhat Disagree; E. Moderately Disagree; F. Strongly Disagree
4. It was important for you to choose a residency program that had opportunities to care for patients from immigrant and refugee populations.
A. Strongly Agree; B. Moderately Agree; C. Somewhat Agree; D. Somewhat Disagree; E. Moderately Disagree; F. Strongly Disagree
5. It was important for you to choose a residency program that had opportunities to care for patients from local low-income and underserved populations.
A. Strongly Agree; B. Moderately Agree; C. Somewhat Agree; D. Somewhat Disagree; E. Moderately Disagree; F. Strongly Disagree
6. It was important for you to choose a residency program that included curriculum on health inequities and social determinants of health.
A. Strongly Agree; B. Moderately Agree; C. Somewhat Agree; D. Somewhat Disagree; E. Moderately Disagree; F. Strongly Disagree
7. Did your residency prepare you to address topics relating to international health, including preparing a patient for international travel, assessing the returned traveler, and international adoption?
A. Strongly Agree; B. Moderately Agree; C. Somewhat Agree; D. Somewhat Disagree; E. Moderately Disagree; F. Strongly Disagree
8. Your residency prepared you to work with poor and underserved communities?
A. Strongly Agree; B. Moderately Agree; C. Somewhat Agree; D. Somewhat Disagree; E. Moderately Disagree; F. Strongly Disagree
9. Your residency prepared you to work with immigrant/refugee populations?
A. Strongly Agree; B. Moderately Agree; C. Somewhat Agree; D. Somewhat Disagree; E. Moderately Disagree; F. Strongly Disagree
10. When you are finished with residency, do you plan on spending any professional time working and caring for patients from any of the following: (select all that apply)
A. Immigrant/refugee community locally; B. International population, abroad; C. Poor and underserved communities (whether locally or abroad); D. None of the above
11. During your residency, did you receive education or training on any of the following topics in international/global health? (select all that apply)
A. Health care of immigrant or refugee children and their families; B. Epidemiology of infant and child mortality in developing countries; C. Socioeconomic determinants of global child health; D. Diagnosis and management of malnutrition in developing countries; E. Diagnosis and management of common pediatric tropical diseases; F. Ethical issues in working or volunteering in developing countries; G. International child health policies, initiatives, and guidelines; H. Health care delivery systems in developing countries; I. Preparation for responding to humanitarian emergencies
Part IV: Interest in Global Health Scholarly Activities
1. Would you be interested in participating in additional activities related to global health during residency?
A. Yes; B. No; C. Unsure
2. If yes, please select your level of interest in the following activities (some currently exist in our program, but some do not):
A. Incorporating international health curriculum into your general lecture series (e.g., noon conferences) I. No interest; II. Minimal interest; III. Strong interest
B. Opportunities to work at a refugee clinic I. No interest; II. Minimal interest; III. Strong interest
C. Global Health Grand Rounds (1-2x per year) I. No interest; II. Minimal interest; III. Strong interest
D. Dedicated interdisciplinary global health track within residency I. No interest; II. Minimal interest; III. Strong interest
E. Online global health courses from residency institution (e.g., Canvas modules) I. No interest; II. Minimal interest; III. Strong interest
F. Online global health courses from outside institutions I. No interest; II. Minimal interest; III. Strong interest
G. Certificate in global health from an outside institution I. No interest; II. Minimal interest; III. Strong interest
H. Coursework counting toward Master's in Public Health I. No interest; II. Minimal interest; III. Strong interest
I. Participating in a quarterly pediatric-focused global health interest group I. No interest; II. Minimal interest; III. Strong interest
J. Global Health Journal Club I. No interest; II. Minimal interest; III. Strong interest
1. If interested in a journal club, what format would you prefer to engage in Global Health Journal Club sessions? I. In-person; II. Virtual; III. Both
2. How often would you prefer to engage in Global Health Journal Club sessions? I. Monthly; II. Every two months; III. 3-4 times per year
3. What topics would you be interested in learning about during Global Health Journal Clubs? (free text)
4. Who would you like to choose the articles/topics for the Global Health Journal Club sessions? (select all that apply) I. Self; II. Peers (Fellows or Residents); II. Attending
K. Global health book club I. No interest; II. Minimal interest; II. Strong interest
L. Global health research I. No interest; II. Minimal interest; III. Strong interest
1. If interested in research, would you be interested in helping to do a retrospective chart review of hospital data from Kenya for a global research project? I. Yes; II. No
2. If yes, please provide email address for follow-up: (Free text)
3. Please rank the barriers that prevent you from pursuing global health opportunities.
A. No barriers, just no interest; B. Time; C. Money; D. Health safety; E. Language barriers; F. Political conflict; G. Lack of mentorship; H. Ethical considerations regarding impact
Part V: Global Health and Child Health Inequities
1. If interested in learning about global health during residency, do you plan to work or volunteer in a low-or middle-income community in the future?
A. Yes, definite plans starting after graduation; B. Very likely to seek a long-term position; C. Very likely to seek short-term volunteer opportunities only; D. Somewhat likely to seek a long-term position; E. Somewhat likely to seek short-term volunteer opportunities only; F. Unlikely; G. Definitely not
2. If interested in learning about global health during residency, do you plan to incorporate global health into your career?
A. Yes; B. No
3. Overall, how would you rate your residency in preparing you for global child health activities?
A. Poor; B. Fair; C. Good; D. Very Good; E. Excellent
4. Overall, how would you rate your residency in preparing you for child health inequities?
A. Poor; B. Fair; C. Good; D. Very Good; E. Excellent
5. Please share any additional comments or reflections on global health in residency: (Free text)
Part VI: Global Health and COVID-19
1. Please compare your current interest in participating in an international elective to that before the COVID-19 pandemic. Since pre-COVID-19, your interest in an international elective has:
A. Increased; B. Decreased; C. Unchanged
2. In general, how has your interest in learning about global health (via news media, formal conferences, courses, etc) changed with the COVID-19 pandemic?
A. Increased; B. Decreased; C. Unchanged
3. How has your interest in advocating for the prevention of the international spread of diseases (COVID-19 and other microbes) changed with the COVID-19 pandemic?
A. Increased; B. Decreased; C. Unchanged
4. How has your interest in advocating for global vaccination of vaccine-preventable illnesses (COVID-19, Hepatitis, Influenza, Measles, and other microbes) changed with the COVID-19 pandemic?
A. Increased; B. Decreased; C. Unchanged
5. How has your interest in learning more about global economic, social, and political disparities and access to health care changed with the COVID-19 pandemic?
A. Increased; B. Decreased; C. Unchanged
6. How has your interest in learning more about global resource distribution and scientific development (PPE, medicines, vaccines) changed with the COVID-19 pandemic?
A. Increased; B. Decreased; C. Unchanged
7. If you had an opportunity to participate on an international elective in 2021, would you go?
A. If yes, why? (select all that apply) I. Long-standing interest in global health; II. Guaranteed elective time during residency to travel; III. Personal vaccine status; IV. Vaccine status of close contacts (family, friends, coworkers); V. Personal Physical Health; VI. Personal Mental Health; VII. Interest in novel and valuable opportunity to learn more about global health in setting of a pandemic; VIII. Strong mentorship; IX. Other (free text)
B. If no, why? (select all that apply)
I. No interest;
II. Time;
III. Money;
IV. Personal vaccine status;
V. Vaccine status of close contacts (family, friends, coworkers);
VI. Personal Physical Health;
VII. Personal Mental Health;
VIII. Public health concerns secondary to variable global vaccine distribution;
IX. Public health concerns secondary to the onset of new COVID-19 strains;
X. Public health concerns secondary to continued international recommendations to limit travel/exposure;
XI. Concern for missing valuable curriculum/training experiences in home residency program;
XII. Language barriers;
XIII. Political conflict;
XIV. Lack of mentorship;
XV. Increased awareness of the supremacy mindset intertwined with global health;
XVI. Other (free text)
8. Before the COVID-19 pandemic, how interested were you in participating in domestic global health (working with refugee/immigrant populations, with the Indian Health Services, with individuals experiencing homelessness)?
A. Very interested;
B. Moderately interested;
C. Somewhat interested;
D. Somewhat disinterested;
E. Very disinterested
9. Please compare your current interest in participating in a domestic global health elective to that before the COVID-19 pandemic. Since pre-COVID-19, has your interest:
A. Increased; B. Decreased; C. Stayed about the same
10. If you had an opportunity to participate in domestic global health now, would you participate?
A. If yes, why? (select all that apply)
I. Long-standing interest in global health;
II. Guaranteed elective time during residency to travel;
III. Personal vaccine status;
IV. Vaccine status of close contacts (family, friends, coworkers);
V. Personal Physical Health;
VI. Personal Mental Health;
VII. Interest in novel and valuable opportunity to learn more about global health in the setting of a pandemic;
VIII. Strong mentorship;
IX. Other (free text)
B. If no, why? (select all that apply)
I. No interest;
II. No known opportunities;
III. Time;
IV. Money;
V. Personal vaccine status;
VI. Vaccine status of close contacts (family, friends, coworkers);
VII. Personal physical health;
VIII. Personal mental health;
IX. Public health concerns secondary to variable national vaccine distribution;
X. Public health concerns secondary to the onset of new COVID-19 strains;
XI. Public health concerns secondary to continued recommendations to limit national travel/exposure;
XII. Concern for missing valuable curriculum/training experiences in regular clinical rotations;
XIII. Language barriers;
XIV. Lack of mentorship;
XV. Other (free text)
11. To what degree do you think your global health education has been adversely impacted by the COVID-19 pandemic?
A. Significant adverse impact; B. Moderate adverse impact; C. Slight adverse impact; D. No impact at all; E. Positively impacted
To what degree do you think your overall scholarly activity has been adversely impacted by the COVID-19 pandemic?
A. Significant adverse impact;
B. Moderate adverse impact;
C. Slight adverse impact;
D. No impact at all;
E. Positively impacted
13. To what degree do you think your overall residency education has been adversely impacted by the COVID-19 pandemic?
A. Significant adverse impact;
B. Moderate adverse impact;
C. Slight adverse impact;
D. No impact at all;
E. Positively impacted
Part VII: Demographics
1. What is your level of training?
A. PGY1; B. PGY2; C. PGY3; D. PGY4+
3. What specialty are you doing your training in?
A. Categorical Peds; B. Med/Peds; C. EM/Peds; D. Peds/Psych/Child Psych; E. Child Neurology; F. Neurodevelopmental Peds
3. Where did you attend medical school?
A. United States; B. Canada; C. Other
4. Did you graduate from an MD or DO medical program?
A. MD; B. DO; C. Other
5. What is your approximate level of educational debt? (including your spouse's)
A. < $100,000; B. > $100,000
6. What are your plans for clinical practice in the future?
A. Primary care practice; B. Subspecialty practice/both primary and subspecialty practice; C. Hospitalist; D. Other
7. What gender do you identify with?
A. Male; B. Female; C. Transgender; D. Gender fluid; E. Non-binary; F. Other; G. Prefer not to answer
8. What race/ethnic group do you most identify with?
A. White, non-Hispanic; B. Asian, Southeast Asian, Pacific Islander; C. Black; D. Hispanic; E. Other
9. What is your age in years?
A. 20-25; B. 26-30; C. 31-35; D. 36-40; E. 40+
10. Are you married/partnered?
A. Yes; B. No
11. Do you have any children (or currently expecting)?
A. Yes; B. No
12. How many languages do you speak and/or understand fluently?
A. 1 language; B. 2 languages; C. 3 languages; D. 3+ languages
Part VIII: Graduating Question
1. Please fill in your name and email address if you would like to receive a $5 Amazon gift card. (Free text)

**Table 3:** Supplemental Content 2. Increased, Decreased, or Neutral Changes in the Interest of Global Health Topics Following COVID-19 (N=96)

Effect of the Pandemic on Interest Related to Global Health Topics and Electives	Increased, N (%)	Unchanged, N (%)	Decreased, N (%)
Participating in a domestic global health elective when compared to before the COVID-19 pandemic	18 (19)	76 (79)	2 (2)
Participating in an international elective when compared to before the COVID-19 pandemic	10 (10)	65 (68)	21 (22)
Learning about global health, specifically because of the COVID-19 pandemic	26 (27)	68 (71)	2 (2)
Learning about global resource distribution and scientific development	52 (54)	44 (46)	0 (0)
Awareness of global economic, social, and political disparities and access to healthcare	49 (51)	47 (49)	0 (0)
Advocating for global vaccination against vaccine-preventable illnesses	62 (65)	34 (35)	0 (0)
Advocating for preventing the international spread of diseases	52 (54)	44 (46)	0 (0)
